# Editorial: Evidence-based frameworks of assessment and treatment in forensic psychiatry practice

**DOI:** 10.3389/fpsyt.2023.1301759

**Published:** 2023-10-13

**Authors:** Yasin Hasan Balcioglu, Fatih Oncu, Conor O'Neill, Gautam Gulati, Nicholas Scurich

**Affiliations:** ^1^Forensic Psychiatry Unit, Department of Psychiatry, Bakirkoy Prof. Mazhar Osman Training and Research Hospital for Psychiatry, Neurology, and Neurosurgery, Istanbul, Turkiye; ^2^Department of Psychiatry, Trinity College Dublin, Dublin, Ireland; ^3^School of Law, University of Limerick, Limerick, Ireland; ^4^Departments of Psychological Science and Criminology, Law and Society, University of California, Irvine, Irvine, CA, United States

**Keywords:** forensic psychiatry, forensic psychiatric care, violence, offender, recidivism, risk assessment, violent radicalization, female offender

Forensic psychiatry thrives within the complex overlap of two seemingly distinct realms: the legal arena and the field of psychiatry. It focuses on evaluating and treating mental disorders when they appear linked to criminal behavior. Forensic psychiatry has evolved into a recognized discipline with a robust background of scientific inquiry, primarily because mental health care has always had a significant interface with the law. While it is fundamentally a clinical discipline, it also demands a comprehensive understanding of the law for effective clinical practice.

While most instances in this field generally do not generate significant conflicts, the intersection of medicine and law can occasionally give rise to conflict and intricate ethical dilemmas, sometimes concurrently, due to its inherent subjectivity (1). To effectively address these issues, forensic psychiatry should firmly establish itself on the foundation of academic rigor and an evidence-based approach within its own domain. Whether engaging in psychiatric assessments or treatments, the practice of forensic psychiatry should be underpinned by empirical data and statistical analysis. Despite variations in legal frameworks governing forensic psychiatry across different countries and jurisdictions, it is crucial to prioritize the incorporation of the most robust available scientific knowledge into the practice of the field (2). This effort not only contributes to the pursuit of justice but also holds the potential to mitigate reoffending and advance broader objectives related to public safety and health.

The assessment of violence risk is one of the most extensively researched areas in this field (3). Currently, over 400 structured instruments have been developed in forensic psychiatry and the criminal justice system to assess the risk of violence and offending (4). Structured risk assessment tools with robust scientific validity are of significant importance within the field of forensic psychiatric practice for describing risk factors and their relationship to violence (5, 6). These tools find widespread application in shaping initial sentencing, parole determinations, and choices related to post-release supervision and rehabilitation. These tools also play a central role in mental health services, particularly within forensic mental health services, by systematically allocating patients to the appropriate levels of therapeutic security (7).

Conducting a scientific evaluation of current forensic psychiatric practices serves as a sound foundation for creating forensic assessments that are attuned to the intricacies of real-world forensic practice. Additionally, it provides a basis for developing effective treatment approaches aimed at reducing reoffending. This Research Topic seeks to contribute evidence-based and innovative insights to the existing body of knowledge and practices in forensic psychiatry research. It also aims to introduce the latest scientific information pertinent to the assessment and treatment within the field of forensic psychiatry.

In the study conducted by Skrivánková et al., the aim was to utilize the Structured Assessment of Violence Risk in Youth (SAVRY) tool in their adolescent sample in Czechia to assess violence risk, protective factors, and personality variables associated with these factors, thereby facilitating precise targeting for therapeutic interventions. Their findings revealed that the most robust protective factor, irrespective of gender or a history of violence, was Strong Attachment and Bonds (Factor P3). Additionally, they identified three internal factors within the SAVRY tool—social conduct, assimilation, and maladaptation, through factor analysis. Some of the personality traits and maladaptive strategies including intrapsychic tension, sensitivity to peer rejection, and anxiety are associated with violence risk. They concluded that identifying risk, protective factors, and personality variables and focusing adaptive strategies of young people are crucial for evaluating and designing appropriate interventions for violent juveniles. Some personality traits and maladaptive strategies, such as intrapsychic tension, sensitivity to peer rejection, and anxiety, are associated with the risk factors of violence. The conclusion drawn from this study is that it is crucial to identify risk and protective factors, along with personality variables, and to concentrate on developing adaptive strategies for young individuals when assessing and designing appropriate interventions for violent juveniles.

An essential component of forensic mental healthcare pertains to informal social networks, encompassing connections with family, friends, peers, and romantic partners. A randomized controlled trial by Swinkels et al. has addressed the effectiveness of a evidence-based social network intervention for forensic psychiatric patients in Netherlands. This study included 102 forensic psychiatric outpatients receiving treatment as usual (TAU) and compared two groups by allocating them to either only TAU or TAU plus an additive informal social network intervention. Follow-up assessments were conducted at 3, 6, 9, 12, and 18 months after baseline. The findings revealed that participants who only received TAU were hospitalized 2.1 times more frequently within 12 months and 4.1 times more frequently within 18 months. Additionally, they reported an average of 2.9 times more criminal behaviors over time compared to participants who received the additive intervention. This study underscores the effectiveness of optimizing forensic outpatient treatment by fostering collaboration with informal care initiatives designed to enhance social networks within the community, resulting in reduced hospitalization and criminal behavior.

This topic also encompasses research that addresses the factors associated with criminal recidivism, represented by two notable papers from Scandinavia. In their retrospective register-based cohort study, Sivak et al. examined the influence of key criminological and demographic variables on treatment duration and explored its impact on criminal recidivism after discharge from care in a sample of forensic psychiatric patients from Sweden. The duration of treatment was longer for individuals who had committed violent crimes, suffered from psychosis, or had a history of substance use disorder, as well as for those whose sentences involved special court supervision. They found that the cumulative incidence of violent crime post-discharge was approximately six percent at 12 months and around ten percent at 24 months. In addition, the results of this study revealed that in patients without a history of substance use disorder and patients whose sentences did not include special court supervision, recidivism was significantly higher in those with a shorter treatment duration. In another registry-based study among a Finnish forensic psychiatric sample, Ojansuu et al. examined recidivism risk for over 500 patients released from forensic care between 1999 and 2018, with a follow-up time of up to 20 years, and aimed to reveal the factors associated with an increased risk of general and violent recidivism. The study observed a general recidivism rate of 2 015 per 100 000 person-years and a violent recidivism rate of 1 083 per 100 000 person-years. Notably, the findings suggest a significant inverse correlation between the duration of treatment and the risk of general recidivism. Additionally, male gender, the presence of a comorbid substance use disorder, and younger age at the time of discharge were identified as factors significantly associated with a increased risk of recidivism. Both studies underscored the potential effect of longer periods of forensic inpatient care in reducing the risk of criminal recidivism.

In the realm of forensic psychiatric research, which has traditionally centered on male offenders, there exists a notable gap in the exploration of female offenders, marking this as an understudied yet significant area warranting further investigation. Trägårdh et al. highlighted the characteristics of Swedish female violent offenders and conducted a comparative analysis between those with and without a severe mental disorder (SMD). Regardless of their SMD diagnosis, female offenders of lethal and severe violence had a high prevalence of previous violent victimization. Subjects without SMD were more likely to exhibit patterns of anxiety, personality disorders, and substance use disorders. They also had a higher incidence of prior criminal records, faced charges of lethal index violence more frequently, and were more likely to have male adult intimate partners or ex-intimate partners as victims who had abused the offender. Additionally, both the offender and the victim were more often found to be under the influence of a substance. This study underscores that female offenders of lethal and severe violence exhibit distinctive characteristics when considering the presence of SMD in terms of background features and the victim-perpetrator context. It also emphasizes the necessity of developing nuanced interventions to meet their rehabilitative needs while simultaneously addressing community protection, underscoring the need for further research in this area.

Recently, violent extremism has gained specific attention as a topic in the field of forensic psychiatry and necessitates a distinct approach compared to other forms of violent offenses due to differences in ideologies, attitudes, grievances, motivations, intentions, backgrounds, objectives, criminal histories, and mental health profiles. Violent extremism risk assessments, relevant for legal decisions, prison and probation settings, and inter-professional risk collaboration, require reports adaptable to various judicial contexts, and evaluations, including practitioner feedback and standardized assessments, to gauge their quality and utility. Violent Extremism Risk Assessment tool (VERA) and its revised version, VERA-2R, provide evidence-based assessments rooted in empirical and expert knowledge of radicalization, violent extremism, and terrorism. They rely on evaluators' capacity to gain meaningful insights into an individual's risk propensity. Duits et al. surveyed forensic professionals in three European countries to explore VERA-2R usage in various judicial contexts and assess the importance of organizational aspects. The study found that professionals see value in the VERA-2R for information organization and common risk language but encounter challenges related to limited usage opportunities, insufficient support, and organizational collaboration issues. In another study by Duits and Kempes, it was aimed to assess the reliability of the VERA-2R, with a specific focus on both interrater and intrarater reliability. According to the results, the level of agreement on indicators and structured risk assessments, as revealed in both interrater and intrarater reliability, can be categorized as strong, demonstrating the importance of this study in investigating the reliability of a Structured Professional Judgment tool with trained assessors using judicial files as a basis.

Overall, this Research Topic encompasses a wide range of research within forensic psychiatry, addressing current and pivotal subjects, including violence risk assessment, risk factors for recidivism, characteristics of female offenders, and violent extremism. The published papers emanate from geographically varied regions, effectively catering to a international audience. The topic reinforces the critical necessity for continued international research endeavors to advance scholarly knowledge and contribute substantively to the existing body of literature, thereby holding potential implications for research methodologies, clinical practices, and policy formulation within the domain of forensic psychiatry.

## Author contributions

YB: Writing—original draft. FO: Writing—review and editing. CO'N: Writing—review and editing. GG: Writing—review and editing. NS: Writing—review and editing.
